# Supplementary material for: Functional brain activity constrained by structural connectivity reveals cohort-specific features for serum neurofilament light chain
Source: Commun Med (Lond). 2022 Jan 17;2:8. doi: 10.1038/s43856-021-00065-5 (PMC9053240; doi:10.1038/s43856-021-00065-5)
Supplement: Supplementary file 2 — Supplementary Materials [file 43856_2021_65_MOESM2_ESM.pdf]

Supplementary Information

Functional Brain Signals Constrained by Structural Brain Connectivity Reveal Cohort Specific Features for Serum Neurofilament Light Chain

Authors

Saurabh Sihag (1), Sébastien Naze (2,3), Foad Taghdiri (4), Melisa Gumus (4), Charles Tator (4,5,6), Robin Green (4,5,7), Brenda Colella (4), Kaj Blennow (8,9), Henrik Zetterberg (8,9,10,11), Luis Garcia Dominguez (12,13), Richard Wennberg (4,5,12), David J. Mikulis (4,5,14), Maria C. Tartaglia (4,5,12), James R. Kozloski (2,#)

Affiliations

1 University of Pennsylvania, Philadelphia, PA, USA;
2 T.J. Watson IBM Research Center, Health Care and Life Sciences, Yorktown Heights, NY, USA;
3 IBM Research Australia, Melbourne, Victoria, Australia;
4 Institute of Medical Science, University of Toronto, Toronto, Canada;
5 Canadian Concussion Centre, University Health Network, Toronto, Canada;
6 Division of Neurosurgery, University Health Network, Toronto, Canada;
7 Department of Rehabilitation Sciences, University of Toronto, Toronto, Canada;
8 Institute of Neuroscience and Physiology, Department of Psychiatry and Neurochemistry, The Sahlgrenska Academy at the University of Gothenburg, Mölndal, Sweden;
9 Clinical Neurochemistry Laboratory, Sahlgrenska University Hospital, Mölndal, Sweden;
10 Department of Neurodegenerative Disease, UCL Institute of Neurology, Queen Square, London, UK;
11 UK Dementia Research Institute at UCL, University College London, London, UK;
12 Division of Neurology, University Health Network, Toronto, Canada;
13 Brain and Behaviour Program, The Hospital for Sick Children, Toronto, Ontario, Canada;
14 Division of Neuroradiology, University Health Network, Toronto, Canada.

# corresponding author: kozloski@us.ibm.com

Supplementary Note 1: FMRIPrep Processing Details

Results included in this manuscript come from preprocessing performed using FMRIPREP version stable[1, 2], a Nipype[3,4] based tool. Each T1w (T1-weighted) volume was corrected for INU (intensity non-uniformity) using N4BiasFieldCorrection v2.1.0[5] and skull-stripped using antsBrainExtraction.sh v2.1.0 (using the OASIS template). Brain surfaces were reconstructed using recon-all from FreeSurfer v6.0.1[6], and the brain mask estimated previously was refined with a custom variation of the method to reconcile ANTs-derived and FreeSurfer-derived segmentations of the cortical gray-matter of Mindboggle[21]. Spatial normalization to the ICBM 152 Nonlinear Asymmetrical template version 2009c[7] was performed through nonlinear registration with the antsRegistration tool of ANTs v2.1.0[8], using brain-extracted versions of both T1w volume and template. Brain tissue segmentation of cerebrospinal fluid (CSF), white-matter (WM) and gray-matter (GM) was performed on the brain-extracted T1w using fast[17].
Functional data was motion corrected using mcflirt (FSL v5.0.9[9]). "Fieldmap-less" distortion correction was performed by co-registering the functional image to the same-subject T1w image with intensity inverted[13,14] constrained with an average fieldmap template[15], implemented with antsRegistration (ANTs). This was followed by co-registration to the corresponding T1w using boundary-based registration[16] with six degrees of freedom, using bbregister (FreeSurfer v6.0.1). Motion correcting transformations, field distortion correcting warp, BOLD-to-T1w transformation and T1w-to-template (MNI) warp were concatenated and applied in a single step using antsApplyTransforms (ANTs v2.1.0) using Lanczos interpolation.

Physiological noise regressors were extracted applying CompCor[18]. Principal components were estimated for the two CompCor variants: temporal (tCompCor) and anatomical (aCompCor). A mask to exclude signal with cortical origin was obtained by eroding the brain mask, ensuring it only contained subcortical structures. Six tCompCor components were then calculated including only the top 5% variable voxels within that subcortical mask. For a CompCor, six components were calculated within the intersection of the subcortical mask and the union of CSF and WM masks calculated in T1w space, after their projection to the native space of each functional run. Frame-wise displacement[19] was calculated for each functional run using the implementation of Nipype. ICA-based Automatic Removal of Motion Artifacts (AROMA) was used to generate aggressive noise regressors as well as to create a variant of data that is non-aggressively denoised[20].

Many internal operations of FMRIPREP use Nilearn[22], principally within the BOLD-processing workflow. For more details of the pipeline see https://fmriprep.readthedocs.io/en/stable/workflows.html.

Supplementary Note 2: Eigenmodes and spatial variation on graphs

The association of the graph frequencies with the spatial variation of the graph signal can be intuitively explained by the mathematical relationship of the graph frequencies with the total variation (TV) of the spectral components given by

$$\mathrm{TV}_G(v_k, \lambda_k) \triangleq \| 1 - \lambda_k/\lambda_{\max}(\mathbf{A}) \|_1 \| v_k \|_1 ,$$

where $\lambda_{\max}(\mathbf{A})$ is the largest eigenvalue of $\mathbf{A}$ and $\|\cdot\|_1$ is the ℓ_1 norm. Based on this equation, the graph frequency λ_l is said to be higher than λ_k if $\mathrm{TV}_G(v_l, \lambda_l) > \mathrm{TV}_G(v_k, \lambda_k)$ (Ortega et al. 2018). Note that for adjacent matrix $\mathbf{A}$, we have

$$\lambda_k = v_k^T \mathbf{A} v_k = \sum_{i \neq j} A_{ij}\, v_k^i v_k^j ,$$

where v_k^i is the i −th element of the eigenvector v_k. Thus, a large value of λ_k implies smaller variation in the elements associated with connected nodes in the eigenvector corresponding to λ_k, whereas a smaller eigenvalue implies that the entries of its corresponding eigenvector at structurally connected nodes tend to be in different directions. Therefore, lower graph frequencies are associated with larger eigenvalues of the adjacency matrix.

Supplementary Figure 1: Example of low and high graph frequency components of BOLD data from a subject

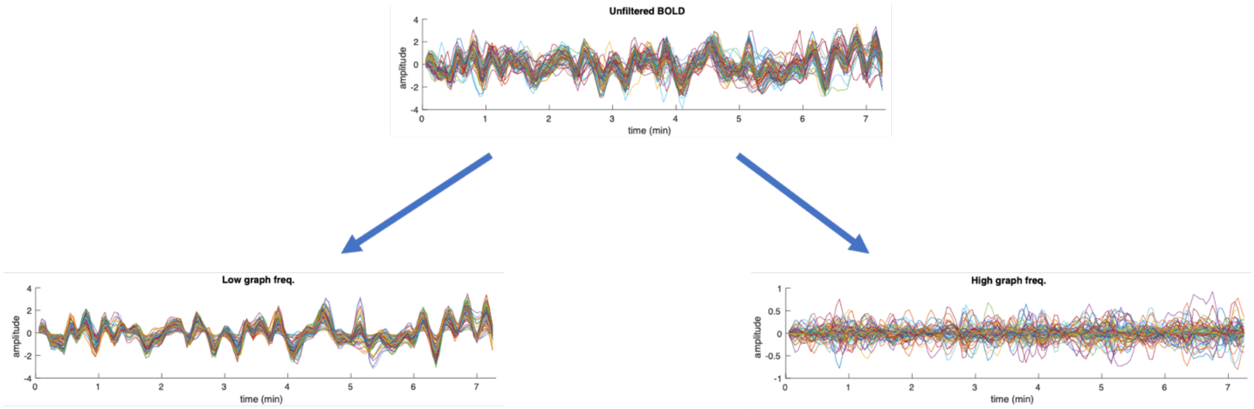

Supplementary Figure 1 *Example of outputs obtained after application of high pass and low pass graph filters on BOLD data from a randomly selected subject. The figures show BOLD time series from the 66 parcellated cortical brain regions with corresponding low and high graph frequencies filtered BOLD signals. For all 66 brain regions, note the smooth spatial evolution in the low pass filtered BOLD time series and highly variable spatial evolution in the high pass filtered BOLD time series.*

Supplementary Note 2

Supplementary Data 18 tabulates the F-scores from linear regression based univariate model selection and Pearson's correlation with serum NfL in the two cohorts. The associated p-values in Supplementary Data 18 have not been corrected for multiple comparisons. After FDR correction, we note that the low graph frequency feature from isthmus cingulate in the right hemisphere had a statistically significant F-score (FDR corrected p-value = 0.0372) in HC cohort. For ExPro cohort, the high graph frequency features from transversetemporal and lingual areas in the left hemisphere had statistically significant F-scores for ExPro cohort but not HC cohort.

The group differences between the correlations with serum NfL for the two cohorts are investigated by evaluating the z-score through the equation:

$$z_i = \frac{\rho_i(HC) - \rho_i(ExPro)}{\sqrt{\frac{1}{(n(HC)-3)} + \frac{1}{(n(ExPro)-3)}}}$$

where $\rho_i(HC)$ is the absolute effect size of correlation for HC cohort and $\rho_i(ExPro)$ is the absolute effect size of correlation for the ExPro cohort, $n(HC)$ is the number of samples in the HC cohort, and $n(ExPro)$ is the number of samples in the ExPro cohort. P-values associated with the z-scores are calculated. Features whose correlations with serum NfL are different in the two cohorts at 0.05 significance level (uncorrected p-values) are highlighted in Supplementary Data 18. Features significant in HC cohort but not in ExPro cohort and having significant group difference are highlighted by blue background. Features significant in ExPro cohort but not in HC cohort with significant group difference are highlighted by yellow background.

Supplementary Note 3

Supplementary Figure 2 includes a carpet plot illustrating the distribution of weights associated with all GSP features included in different PLS models found to have the best prediction performance during leave-one-out cross validation procedure in the HC cohort. For every subject, the 'best' prediction model was chosen from a set of PLS models that were not overfit on the training set consisting of the rest of the subjects at 0.0001 significance level. The PLS models were formed using one component, thus allowing distinct weights to be associated with the input GSP features.

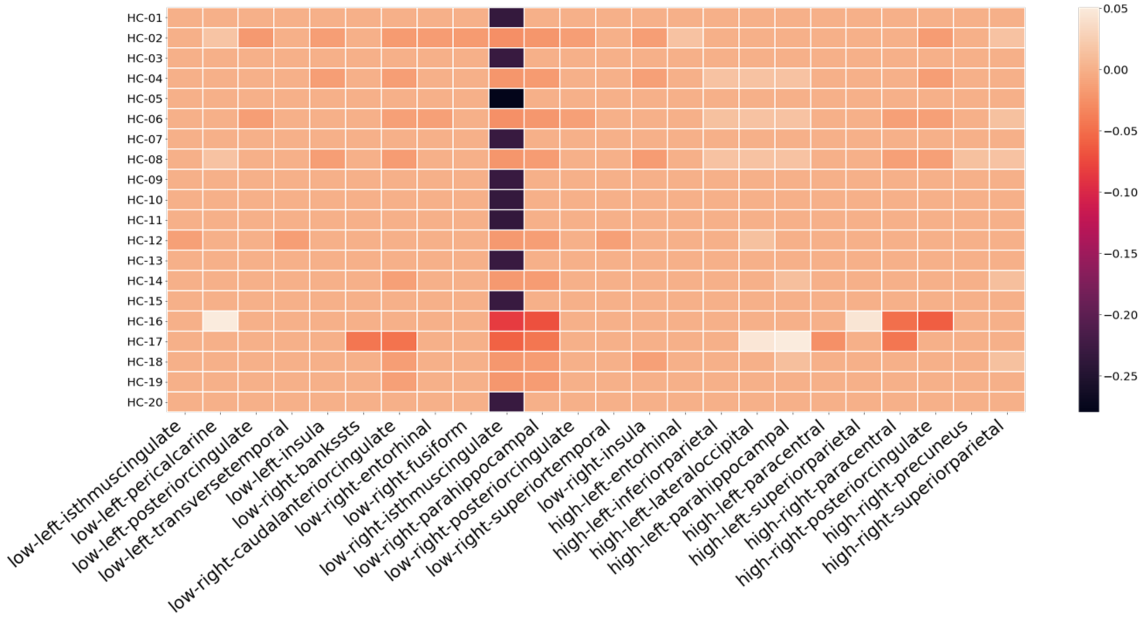

Supplementary Figure 2 *Carpet plot for weights associated with GSP features included in the best performing prediction model for every subject in the leave-one-out cross validation procedure. Each row is associated with a distinct subject (HC-Y corresponds to subject Y) that was used as the test case and the PLS regression model trained using the data from all other subjects. The columns span all GSP features that were selected over the complete cross validation procedure. For every row, the elements of the carpet plot represent the weights associated with selected GSP features for a PLS model that best predicted the serum NfL for the given subject.*

Supplementary Note 4

Supplementary Figure 3 includes a carpet plot illustrating the distribution of weights associated with all GSP features included in different PLS models found to have the best prediction performance during leave-one-out cross validation procedure in the ExPro cohort. For every subject, the 'best' prediction model was chosen from a set of PLS models that were not overfit on the training set consisting of the rest of the subjects at 0.0001 significance level. The PLS models were formed using one component, thus allowing distinct weights to be associated with the input GSP features.

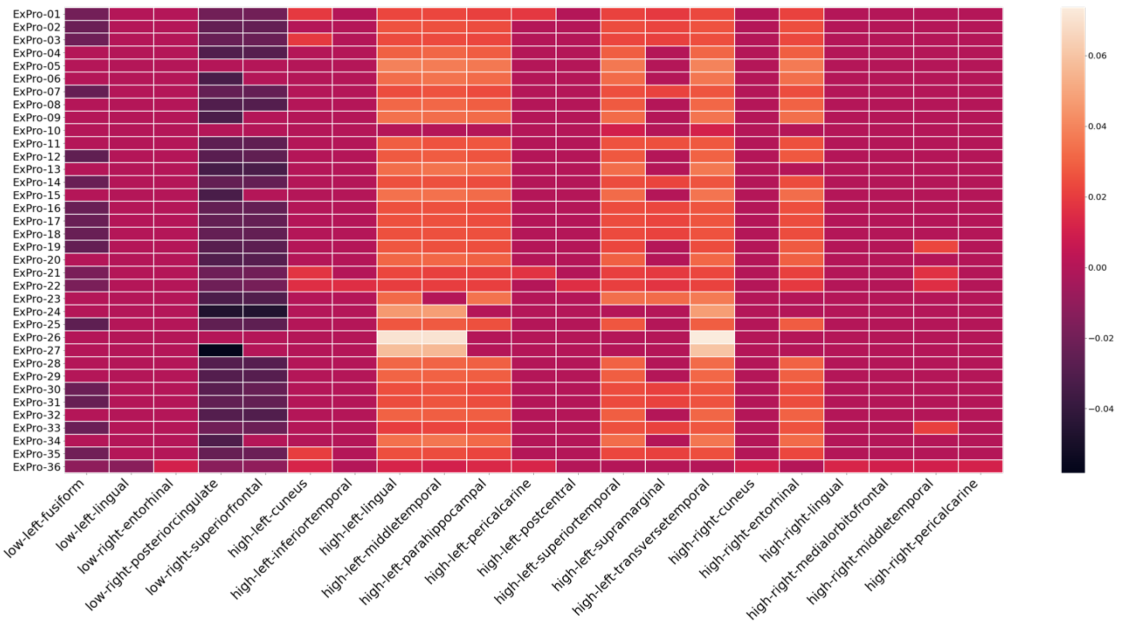

Supplementary Figure 3 *Carpet plot for weights associated with GSP features included in the best performing prediction model for every subject in the leave-one-out cross validation procedure. Each row is associated with a distinct subject (ExPro-Y corresponds to subject Y) that was used as the test case and the PLS regression model trained using the data from all other subjects. The columns span all GSP features that were selected over the complete cross validation procedure. For every row, the elements of the carpet plot represent the weights associated with selected GSP features for a PLS model that best predicted the serum NfL for the given subject.*

Supplementary Note 5: PLSR models built from GSP features explain significant variance in serum NfL levels in the two groups

We adopted a VIP score threshold-based methodology to determine the explanatory performance of GSP features in context of serum NfL. To start with, we had 48 GSP features for HC subjects and 45 GSP features for ExPro subjects that had VIP scores greater than 1 (see Supplementary Data 19). The PLS models with VIP scores greater than 1 were determined to be overfit to the serum NfL levels from the respective groups using non-parametric permutation tests. This was expected due to high multicollinearity among GSP features and potentially the likelihood of a small subset of 132 GSP features explaining significant variance in serum NfL. Therefore, the cutoff on the VIP score to build a model that did not overfit on the serum NfL was expected to be greater than 1 for each cohort (Chong and Jun 2005). Supplementary Figure 4 shows that the null distributions of R^2 of PLSR models with GSP features obtained by different thresholds on the VIP scores of GSP features are distinct for ExPro compared to HC subjects. The results show that there were two models that were not overfit (p-value < 0.001 in permutation test) on serum NfL in the HC cohort. For ExPro cohort, there were 12 models that were not overfit on serum NfL.

Supplementary Figure 4: R^2 for different PLS regression models.

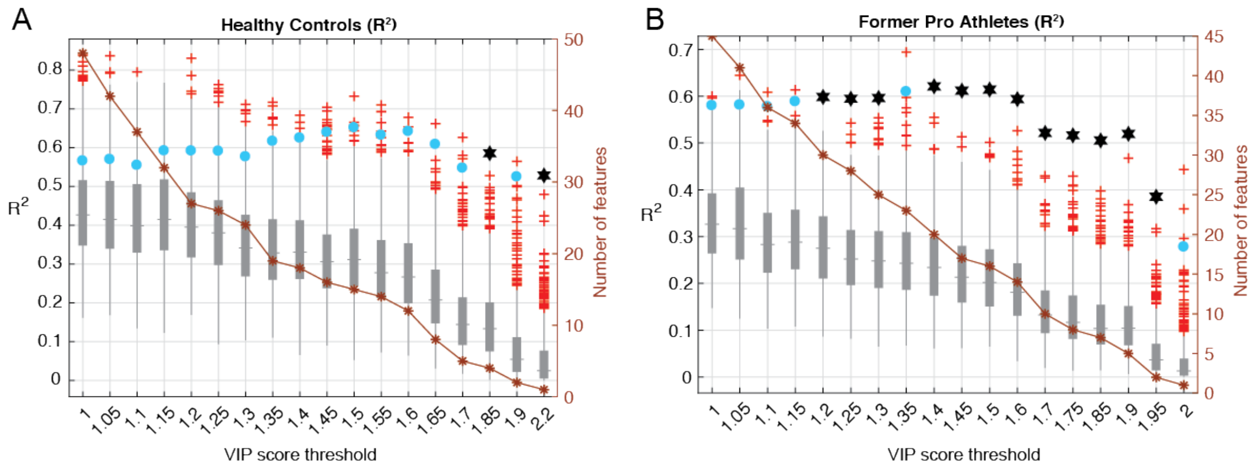

Explained variance R^2 for A) healthy controls and B) former professional athletes derived from sets of GSP features whose variable importance in projection (VIP) scores were greater than the given thresholds. R^2 values were denoted by black stars or blue dots to indicate statistically significant (p-value < 0.001) or insignificant with respect to the null distribution in the non-parametric permutation test of models (gray boxes with whiskers extending to 25th and 75th percentiles). Outliers are represented by red crosses.

Supplementary Figure 5: Brain regions associated with the GSP features in selected PLSR Models for healthy controls (HC) and former athletes with history of concussion (ExPro).

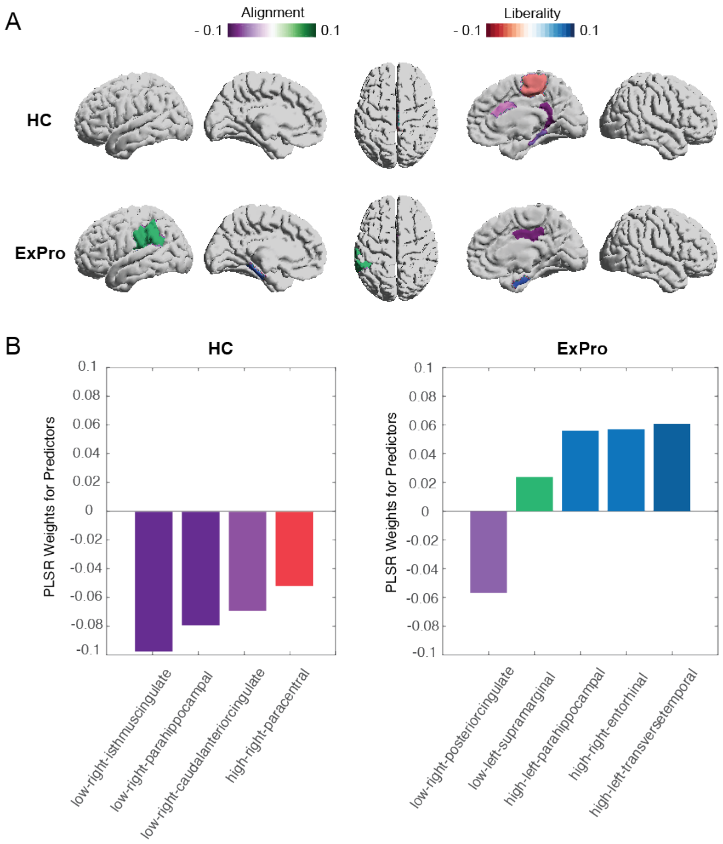

A) Projections onto the cortical surface of brain regions from the Desikan-Killiany atlas that are associated with the GSP features of the PLSR models for healthy controls (HC) and former athletes (ExPro). Color-code denotes "Alignment" and "Liberality" relationships associated to low and high graph frequencies, respectively, for both cohorts of subjects.
B) PLSR weights that constituted the single component in the regression model with GSP features as predictors model are shown explicitly. Color-code is consistent with A).

We illustrate two representative non-overfit PLS models for each cohort in Supplementary Figure 5. The PLS model for HC explained 58.6% of the variance in serum NfL levels (see Supplementary Figure 4). Interestingly, we observed that the GSP feature with the highest VIP score for HC subjects, i.e., low graph frequency feature from isthmus cingulate in the right hemisphere, explained 52.0% of the variance in serum NfL levels for HC subjects. For ExPro subjects, the PLSR model using 5 GSP features (VIP score > 1.9) explained 52.0% variance in serum NfL levels.

We note that the three low graph frequency features for the HC cohort in Supplementary Figure 4B also have the largest association with serum NfL in Fig. 2. Similarly, for the ExPro cohort, all GSP features in Supplementary Figure 4B except for low-left-supramarginal were significantly associated with serum NfL based on univariate feature selection in Fig. 2.

Supplementary Note 6: Non transferability of prediction performance of PLSR models in two cohorts

We evaluated whether the ordering of the GSP features according to their VIP scores for predicting serum NfL levels were consistent for the two groups. We used VIP score-based variable selection methods for building PLSR models in Supplementary Figures 4 and 5. For the GSP features included in Supplementary Figure 5 for the ExPro model, we observed that in the context of the HC group, only the high graph frequency feature from left parahippocampal area and the low graph frequency feature from the right posterior cingulate had a VIP score greater than 1 (with same sign of correlation with serum NfL levels) and all other features had VIP scores less than 0.6 (see Supplementary Data 19), indicating that most features from the ExPro model were unlikely to be relevant for predicting serum NfL levels for the HC group. Similarly, for the HC model in Supplementary Figure 5, in the context of ExPro group, we observed that only the low graph frequency feature from the right isthmus cingulate area had a VIP score greater than 1 (with same sign of correlation with serum NfL levels) and all other features had a VIP score less than 0.6, which indicates that most features from the HC model were unlikely to be relevant for predicting serum NfL levels in ExPro group. Therefore, these observations imply that distinct GSP features are relevant for explaining the variation in serum NfL levels in the two groups and that therefore differences in the structure-function relationships among brain regions for the two groups result in different models for predicting serum NfL levels.

Supplementary Table 1: Statistics for mediation analysis between age and serum NfL with GSP feature from right entorhinal as mediator variable for ExPro subjects.

	Coefficient	Std. error	p-value
Path a	0.03	0.02	0.0371
Path b	1.57	0.72	0.0055
Path c' (adjusted effect)	0.18	0.04	2.5e-5
Mediation (a*b)	0.05	0.03	0.0299
Path c (total effect) (Age -> serum NfL)	0.2292	0.055	2.58e-5

All paths and the mediation effect of the GSP feature from entorhinal area were statistically significant (p-value < 0.05) and the coefficient for path 'c' was smaller than the total, unmediated effect between age and serum NfL. These observations indicate that the high graph frequency feature from entorhinal in right hemisphere partially mediated the effect of age on serum NfL in ExPro subjects.

Supplementary Table 2: ***Statistics for mediation analysis between age and serum NfL with GSP feature from left transverse temporal as mediator variable for ExPro subjects.***

	Coefficient	Std. error	p-value
Path a	0.0232	0.016	0.0186
Path b	1.809	1.221	0.0131
Path c' (adjusted effect)	0.1845	0.0496	1.622e-4
Mediation (a*b)	0.0438	0.0367	0.0416
Path c (total effect) (age -> serum NfL)	0.229	0.055	2.387e-5

All paths and the mediation effect of the GSP feature from transverse temporal area were statistically significant (p-value < 0.05) and the coefficient for path 'c' was smaller than that for total, unmediated effect between age and serum NfL. These observations indicate that the high graph frequency feature from transverse temporal in left hemisphere partially mediated the effect of age on serum NfL in ExPro subjects.

Supplementary Note 7: Aging-related GSP features trained on ExPro group predict serum NfL levels in HC group

Neurofilament rises with aging (Khalil et al. 2018) but is also a marker of neurodegeneration and concussion. In this study, age was found to be significantly correlated with the serum NfL level for both the HC group (Pearson's correlation = 0.71, p-value = 4.13×10^{-4}) and the ExPro group (Pearson's correlation = 0.552, p-value = 4.8×10^{-4}).

Analysis of a linear regression model between age and NfL revealed that for the HC group, age explained 50.89% of the variance (R^2 value) in serum NfL levels and predicted 39.2% of the variance (Q^2 value) in serum NfL levels (evaluated based on leave-one-out cross validation). For the ExPro group, age explained 30.45% of the variance (R^2 value) in serum NfL levels and predicted 22.59% of the variance (Q^2 value) in serum NfL levels (evaluated based on leave-one-out cross validation). Furthermore, the prediction model with age as a predictor and trained on the HC group predicted 28.0% of the variance in serum NfL levels of the ExPro group. The prediction model with age as a predictor and trained on the ExPro group predicted 48.2% of the variance in serum NfL levels of the HC group. The better performance of ExPro group in prediction of serum NfL of the HC group in comparison with that for HC group in prediction of serum NfL of the ExPro group could be attributed to the difference in heterogeneity of the two datasets. Specifically, the ExPro group was expected to be more heterogeneous than the HC group, as not all former athletes may be suffering from repercussions of TBI and therefore, the model trained on ExPro group was expected to have a better generalization to serum NfL data from HC group.

In the next set of experiments, we focused on the GSP features that had a significant correlation with age in both groups. We observed that age was significantly correlated (p-value < 0.05) with the high graph frequency feature from the right caudal anterior cingulate and low graph frequency features from right isthmus cingulate and posterior cingulate in both groups. Recall that the low graph frequency feature from right isthmus cingulate was linked with serum NfL for HC subjects and was shown to have partial mediation impact on the relationship of age and serum NfL in this cohort. In contrast, in ExPro subjects, we observed that this GSP feature had a weaker association with serum NfL as compared to HC subjects ($\rho = -0.29$, p-value = 0.0856) and did not have a mediating effect on association between age and serum NfL in ExPro cohort. Also, low graph frequency feature from posterior cingulate was linked with serum NfL for ExPro subjects. This GSP feature had a similar correlation with age for the two groups ($\rho = -0.4945$, for HC subjects and $\rho = -0.4412$ for ExPro cohort). The high graph frequency feature from right caudal anterior cingulate had similar correlation with age for the two groups ($\rho = -0.4992$, for HC subjects and $\rho = -0.4352$ for ExPro cohort) but not a significant correlation with serum NfL for either cohort. Interestingly, the three GSP features also had cohort-specific associations among themselves. Specifically, the high graph frequency feature from caudal anterior cingulate had a significant correlation with the low graph frequency feature from posterior cingulate area ($\rho = 0.5756$, $p -$ value = 0.099) for the HC cohort when controlled for age. However, a similar observation was not observed for the ExPro cohort. For both the HC and ExPro cohorts, we observed that the low graph frequency features from the isthmus cingulate and posterior cingulate were significantly correlated ($\rho = 0.57$ for HC and $\rho = 0.607$ for ExPro, p-value < 0.05 for both) with age as a covariate, and this correlation may be driven by the proximity between these two areas. From these observations, we hypothesize that the high graph frequency feature from caudal anterior cingulate was less likely to be a part of the model with comparable serum NfL prediction performances in the two groups as compared to the other GSP features discussed in this section.

A combination of these features could lead to up to 7 potential models for predicting serum NfL levels. We evaluated the performances of all potential models. We evaluated linear regression models formed by different combinations of the three GSP features by training them on one group and testing them to predict the serum NfL levels of the other group. The corresponding R^2 and Q^2 values for these different models are summarized in Supplementary Table 3 for models trained on data from the ExPro group and Supplementary Table 4 for models trained on data from the HC group. The difference in the isthmus cingulate in its association with serum NfL levels in the two groups is apparent in the results in Supplementary Tables 3 and 4. As expected, we observed that the low graph frequency feature from isthmus cingulate is relevant for explaining the variance in serum NfL levels in the HC group ($R^2 = 52.8\%$), however, this model does not have a comparable performance for predicting the serum NfL levels in the ExPro group. Interestingly, the predictive model with the low graph frequency feature from the isthmus cingulate as a predictor trained on the ExPro group explains only 8.4% variance in the serum NfL levels from the ExPro group while predicting 35.9% of the variance in serum NfL levels from HC subjects. This observation further affirms two main observations made previously. Firstly, the isthmus cingulate is relevant for prediction of serum NfL levels in the HC group but not for ExPro group. Secondly, similar to aging related observations, the heterogeneity in the ExPro group was likely to contribute to the better prediction of serum NfL of HC group by the model trained on features from ExPro group as compared to the prediction performance on serum NfL of the ExPro group by the model trained on the features of the HC group. Furthermore, our experiments indicated that the discrepancy associated with isthmus cingulate region was highly unlikely to be random. When the complete dataset was randomly shuffled 1000 times and 36 subjects from the two groups were used in the training dataset and the rest for testing, we observed that the prediction performance of 35.86% on healthy controls was higher than the predicted variance of a random test dataset for 99.95% of the experiments. On the other hand, the explained variance of 52.85% for former athletes was smaller than the explained variance of a random training dataset for 92.8% of the experiments. Furthermore, the joint observation that the explained variance was lower than that for former athletes and the predicted variance was higher than that for healthy controls was observed in only 0.2% of the experiments (p-value = 0.002). These results indicated that the difference in the performance of low graph frequency feature from isthmus cingulate to predict serum NfL levels in former athletes versus healthy controls was highly unlikely to be random.

We also observed that the high graph frequency feature from the right caudal anterior cingulate was associated with aging but was not relevant for prediction of serum NfL levels in both groups. These observations indicated that even for the individual GSP features that were associated with aging in both groups, there were significant variations in their associations among themselves and with serum NfL levels across individual groups.

Features	R^2 (ExPro)	Q^2 (HC)
iCg	8.4	35.9
pCg	24.8	14.6
caCg	2.9	6.9
iCg + caCg	10.7	37.8
iCg + pCg	25.2	5.2
caCg + pCg	25.5	12.2
iCg + pCg + caCg	25.9	4.1

Supplementary Table 3. *Performances of different regression models trained on data from ExPro subjects with serum NfL levels as response and tested on HC subjects. Features: low graph frequency feature from isthmus cingulate in the right hemisphere (iCg), low graph frequency feature from posterior cingulate in the right hemisphere (pCg), high graph frequency feature from caudal anterior cingulate in right hemisphere (caCg).*

Features	R^2 (HC)	Q^2 (ExPro)
iCg	52.9	5.8
pCg	16.2	23.0
caCg	8.0	1.9
iCg + caCg	52.9	2.8
iCg + pCg	54.4	0.2
caCg + pCg	16.2	23.1
iCg + pCg + caCg	55.6	-0.2

Supplementary Table 4. *Performances of different regression models trained on data from HC subjects with serum NfL levels as response and tested on ExPro subjects. Features: low graph frequency feature from isthmus cingulate in the right hemisphere (iCg), low graph frequency feature from posterior cingulate in the right hemisphere (pCg), high graph frequency feature from caudal anterior cingulate in right hemisphere (caCg).*

Supplementary Note 8: Mediation analysis for cortical thickness of pericalcarine, serum NfL and low frequency feature from right isthmus cingulate in HC subjects

The significant correlation of serum NfL with pericalcarine thickness and low graph frequency feature from the isthmus cingulate in HC subjects motivated us to explore the mediation effect of low graph frequency feature from the isthmus cingulate. We conducted mediation analysis with the thickness of pericalcarine region as the independent variable, serum NfL as the dependent variable, and the low graph frequency feature from the isthmus cingulate as mediator variable.

Supplementary Table 5 lists the statistics from mediation analysis.

	Coefficient	Std. error	p-value (uncorrected)
Path a	2.657	1.533	0.0474
Path b	-2.843	0.9058	1.49e-7
Path c'<br>(adjusted effect)	-9.334	3.86	0.0267
Mediation (a*b)	-6.914	3.79	0.037
Path c<br>(total effect)<br>(pericalcarine thickness -> serum NfL)	-16.15	5.348	0.004

***Supplementary Table 5**. Statistics for mediation analysis between cortical thickness of pericalcarine and serum NfL with GSP feature from right isthmus cingulate as mediator variable for HC subjects.*

From the statistics of mediation analysis, we observe that the total, unmediated effect is larger than the mediated effect of pericalcarine thickness on serum NfL and the coefficients for all paths evaluated are statistically significant at 0.05 level (uncorrected p-value) and imply partial mediation effect.

Supplementary Note 9: Mediation analysis for age and low graph frequency feature from posterior cingulate with left choroid plexus volume as a mediator in ExPro subjects

Volume of left choroid plexus had a significant positive correlation with age ($\rho = 0.4703$, p-value = 0.0038) and NfL ($\rho = 0.3922$, p-value = 0.0177) for ExPro subjects but no correlation for HC subjects. Also, the low graph frequency feature from posterior cingulate in the right hemisphere had a significant negative correlation with the volume of left choroid plexus for ExPro subjects ($\rho = -0.4626$, uncorrected p-value = 0.0045, FDR corrected p-value = 0.0315).

	Coefficient	Std. error	p-value
Path a	15.134	3.956	3.71e-3
Path b	-9.17e-4	3.935e-3	0.0415
Path c' (adjusted effect)	-0.0249	0.0128	0.0838
Mediation (a*b)	-0.0139	0.0068	0.0364
Path c (total effect) (age -> pCg GSP feature)	-0.0389	0.0117	0.0049

Supplementary Table 6. *Statistics for mediation analysis between age and GSP feature from posterior cingulate with volume of left choroid plexus as mediator variable for ExPro subjects.*

The low graph frequency feature from posterior cingulate in the right hemisphere had a significant association with age in this cohort. Therefore, we explored whether any of these factors had a causal association among themselves. We hypothesized age to be the predictor for changes in left choroid plexus volume and GSP features. The mediation analysis with the left choroid plexus volume as the mediator and the low graph frequency feature from posterior cingulate as the dependent variable revealed significant partial mediation effect (as summarized by the statistics in Supplementary Table 6).

Supplementary Note 10: Low graph frequency feature from caudal anterior cingulate is associated with cortical thickness of postcentral in ExPro subjects

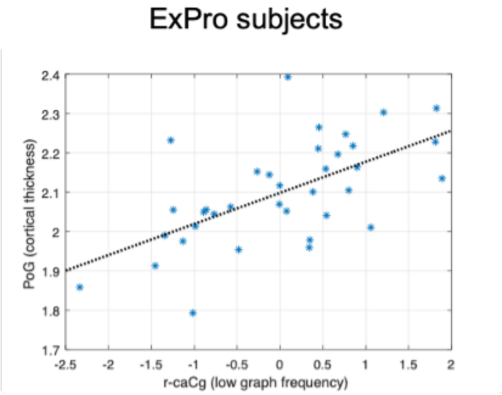

Supplementary Figure 6. *This figure plots mean cortical thickness of postcentral area (PoG) over both hemispheres versus the low graph frequency feature from caudal anterior cingulate (caCg) area in right hemisphere for ExPro cohort.*

Supplementary Figure 6 shows mean cortical thickness of postcentral area over both hemispheres versus the low graph frequency feature from caudal anterior cingulate (caCg) area in right hemisphere for ExPro cohort. This GSP feature was positively correlated with the thickness of postcentral area in both hemispheres for ExPro subjects ($\rho = 0.552, p$-value = 5.36e-4 for right hemisphere and $\rho = 0.5758, p$-value = 2.95e-4 for left hemisphere) when adjusted for age, but not for HC subjects.

Supplementary References

1. Esteban O, Markiewicz CJ, Blair RW, Moodie CA, Isik AI, Erramuzpe A, Kent JD, Goncalves M, DuPre E, Snyder M, Oya H, Ghosh SS, Wright J, Durnez J, Poldrack RA, Gorgolewski KJ. fMRIPrep: a robust preprocessing pipeline for functional MRI. Nat Meth. 2018; doi:10.1038/s41592-018-0235-4

2. fMRIPrep Available from: 10.5281/zenodo.852659.

3. Gorgolewski K, Burns CD, Madison C, Clark D, Halchenko YO, Waskom ML, Ghosh SS. Nipype: a flexible, lightweight and extensible neuroimaging data processing framework in python. Front Neuroinform. 2011 Aug 22;5(August):13. doi:10.3389/fninf.2011.00013.

4. Gorgolewski KJ, Esteban O, Ellis DG, Notter MP, Ziegler E, Johnson H, Hamalainen C, Yvernault B, Burns C, Manhães-Savio A, Jarecka D, Markiewicz CJ, Salo T, Clark D, Waskom M, Wong J, Modat M, Dewey BE, Clark MG, Dayan M, Loney F, Madison C, Gramfort A, Keshavan A, Berleant S, Pinsard B, Goncalves M, Clark D, Cipollini B, Varoquaux G, Wassermann D, Rokem A, Halchenko YO, Forbes J, Moloney B, Malone IB, Hanke M, Mordom D, Buchanan C, Pauli WM, Huntenburg JM, Horea C, Schwartz Y, Tungaraza R, Iqbal S, Kleesiek J, Sikka S, Frohlich C, Kent J,

Perez-Guevara M, Watanabe A, Welch D, Cumba C, Ginsburg D, Eshaghi A, Kastman E, Bougacha S, Blair R, Acland B, Gillman A, Schaefer A, Nichols BN, Giavasis S, Erickson D, Correa C, Ghayoor A, Küttner R, Haselgrove C, Zhou D, Craddock RC, Haehn D, Lampe L, Millman J, Lai J, Renfro M, Liu S, Stadler J, Glatard T, Kahn AE, Kong X-Z, Triplett W, Park A, McDermottroe C, Hallquist M, Poldrack R, Perkins LN, Noel M, Gerhard S, Salvatore J, Mertz F, Broderick W, Inati S, Hinds O, Brett M, Durnez J, Tambini A, Rothmei S, Andberg SK, Cooper G, Marina A, Mattfeld A, Urchs S, Sharp P, Matsubara K, Geisler D, Cheung B, Floren A, Nickson T, Pannetier N, Weinstein A, Dubois M, Arias J, Tarbert C, Schlamp K, Jordan K, Liem F, Saase V, Harms R, Khanuja R, Podranski K, Flandin G, Papadopoulos Orfanos D, Schwabacher I, McNamee D, Falkiewicz M, Pellman J, Linkersdörfer J, Varada J, Pérez-García F, Davison A, Shachnev D, Ghosh S. Nipype: a flexible, lightweight and extensible neuroimaging data processing framework in Python. 2017. doi:10.5281/zenodo.581704.

5. Tustison NJ, Avants BB, Cook PA, Zheng Y, Egan A, Yushkevich PA, Gee JC. N4ITK: improved N3 bias correction. IEEE Trans Med Imaging. 2010 Jun;29(6):1310–20. doi:10.1109/TMI.2010.2046908.

6. Dale A, Fischl B, Sereno MI. Cortical Surface-Based Analysis: I. Segmentation and Surface Reconstruction. Neuroimage. 1999;9(2):179–94. doi:10.1006/nimg.1998.0395.

7. Fonov VS, Evans AC, McKinstry RC, Almli CR, Collins DL. Unbiased nonlinear average age-appropriate brain templates from birth to adulthood. NeuroImage; Amsterdam. 2009 Jul 1;47:S102. doi:10.1016/S1053-8119(09)70884-5.

8. Avants BB, Epstein CL, Grossman M, Gee JC. Symmetric diffeomorphic image registration with cross-correlation: evaluating automated labeling of elderly and neurodegenerative brain. Med Image Anal. 2008 Feb;12(1):26–41. doi:10.1016/j.media.2007.06.004.

9. Jenkinson M, Bannister P, Brady M, Smith S. Improved optimization for the robust and accurate linear registration and motion correction of brain images. Neuroimage. 2002 Oct;17(2):825–41. doi:10.1006/nimg.2002.1132.

10. Andersson JLR, Skare S, Ashburner J. How to correct susceptibility distortions in spin-echo echo-planar images: application to diffusion tensor imaging. Neuroimage. 2003 Oct;20(2):870–88. doi:10.1016/S1053-8119(03)00336-7.

11. Cox RW. AFNI: software for analysis and visualization of functional magnetic resonance neuroimages. Comput Biomed Res. 1996 Jun;29(3):162–73. doi:10.1006/cbmr.1996.0014.

12. Jenkinson M. Fast, automated, N-dimensional phase-unwrapping algorithm. Magn Reson Med. 2003 Jan;49(1):193–7. doi:10.1002/mrm.10354.

13. Huntenburg JM. Evaluating nonlinear coregistration of BOLD EPI and T1w images. Freie Universität Berlin; 2014. Available from: http://hdl.handle.net/11858/00-001M-0000-002B-1CB5-A.

14. Wang S, Peterson DJ, Gatenby JC, Li W, Grabowski TJ, Madhyastha TM. Evaluation of Field Map and Nonlinear Registration Methods for Correction of Susceptibility Artifacts in Diffusion MRI. Front Neuroinform. 2017 [cited 2017 Feb 21];11. doi:10.3389/fninf.2017.00017.

15. Treiber JM, White NS, Steed TC, Bartsch H, Holland D, Farid N, McDonald CR, Carter BS, Dale AM, Chen CC. Characterization and Correction of Geometric Distortions in 814 Diffusion Weighted Images. PLoS One. 2016 Mar 30;11(3):e0152472. doi:10.1371/journal.pone.0152472.

16. Greve DN, Fischl B. Accurate and robust brain image alignment using boundary-based registration. Neuroimage. 2009 Oct;48(1):63–72. doi:10.1016/j.neuroimage.2009.06.060.

17. Zhang Y, Brady M, Smith S. Segmentation of brain MR images through a hidden Markov random field model and the expectation-maximization algorithm. IEEE Trans Med Imaging. 2001 Jan;20(1):45–57. doi:10.1109/42.906424.

18. Behzadi Y, Restom K, Liau J, Liu TT. A component based noise correction method (CompCor) for BOLD and perfusion based fMRI. Neuroimage. 2007 Aug 1;37(1):90–101. doi:10.1016/j.neuroimage.2007.04.042.

19. Power JD, Mitra A, Laumann TO, Snyder AZ, Schlaggar BL, Petersen SE. Methods to detect, characterize, and remove motion artifact in resting state fMRI. Neuroimage. 2013 Aug 29;84:320–41. doi:10.1016/j.neuroimage.2013.08.048.

20. Pruim RHR, Mennes M, van Rooij D, Llera A, Buitelaar JK, Beckmann CF. ICA-AROMA: A robust ICA-based strategy for removing motion artifacts from fMRI data. Neuroimage. 2015 May 15;112:267–77. doi:10.1016/j.neuroimage.2015.02.064.

21. Klein A, Ghosh SS, Bao FS, Giard J, Häme Y, Stavsky E, et al. Mindboggling morphometry of human brains. PLoS Comput Biol 13(2): e1005350. 2017. doi:10.1371/journal.pcbi.1005350.

22. Abraham A, Pedregosa F, Eickenberg M, Gervais P, Mueller A, Kossaifi J, Gramfort A, Thirion B, Varoquaux G. Machine learning for neuroimaging with scikit-learn. Front in Neuroinf 8:14. 2014. doi:10.3389/fninf.2014.00014.
