# Supplementary material for: Functional brain activity constrained by structural connectivity reveals cohort-specific features for serum neurofilament light chain
Source: Commun Med (Lond). 2022 Jan 17;2:8. doi: 10.1038/s43856-021-00065-5 (PMC9053240; doi:10.1038/s43856-021-00065-5)
Supplement: Supplementary file 23 — Reporting Summary [file 43856_2021_65_MOESM23_ESM.pdf]

nature research

Corresponding author(s): James Kozloski

Last updated by author(s): Nov 29, 2021

Reporting Summary

Nature Research wishes to improve the reproducibility of the work that we publish. This form provides structure for consistency and transparency in reporting. For further information on Nature Research policies, see our Editorial Policies and the Editorial Policy Checklist.

Statistics

For all statistical analyses, confirm that the following items are present in the figure legend, table legend, main text, or Methods section.

n/a	Confirmed	
☐	☒	The exact sample size (*n*) for each experimental group/condition, given as a discrete number and unit of measurement
☐	☒	A statement on whether measurements were taken from distinct samples or whether the same sample was measured repeatedly
☐	☒	The statistical test(s) used AND whether they are one- or two-sided<br>*Only common tests should be described solely by name; describe more complex techniques in the Methods section.*
☐	☒	A description of all covariates tested
☐	☒	A description of any assumptions or corrections, such as tests of normality and adjustment for multiple comparisons
☐	☒	A full description of the statistical parameters including central tendency (e.g. means) or other basic estimates (e.g. regression coefficient) AND variation (e.g. standard deviation) or associated estimates of uncertainty (e.g. confidence intervals)
☐	☒	For null hypothesis testing, the test statistic (e.g. *F*, *t*, *r*) with confidence intervals, effect sizes, degrees of freedom and *P* value noted<br>*Give P values as exact values whenever suitable.*
☒	☐	For Bayesian analysis, information on the choice of priors and Markov chain Monte Carlo settings
☒	☐	For hierarchical and complex designs, identification of the appropriate level for tests and full reporting of outcomes
☐	☒	Estimates of effect sizes (e.g. Cohen's *d*, Pearson's *r*), indicating how they were calculated

Our web collection on statistics for biologists contains articles on many of the points above.

Software and code

Policy information about availability of computer code

Data collection	All structural and resting state scans were performed on a 3 Tesla MRI Scanner (GE Signa HDx, Milwaukee, WI, USA) with a standard 8-channel head coil. A high resolution T1-weighted images were obtained using inversion recovery fast spoiled gradient echo (IR-FSPGR), with the following parameters: 180 slices with 1 mm thickness; 3 ms echo time (TE); 7.8 ms repetition time (TR); 450 ms inversion time (TI); 15 flip angle; 25.6 cm field of view (FOV); 256×256 matrix size; 1×1×1 mm^3 voxel size. At least one DWI scan was obtained with diffusion gradients applied across 60 spatial directions (b = 1,000 s/mm^2) as well as 10 non-diffusion weighted (B0) scans. The DWI had the following parameters: 2.4 mm thick axial slices, TR = 17,000 ms, FOV = 23 cm, 2.4×2.4 mm^2 in-plane resolution. Prior to the resting-state functional MRI scan, participants were instructed to close their eyes, not think of anything in particular, and to not fall asleep. The scan acquisition was 5 min 8 s using T2^*-weighted echo-planar imaging with the following parameters: TR = 2,000 ms, TE = 30 ms, 64×64 matrix, 20-cm FOV, flip angle = 85, 40 slices, 3.125×3.125×4 mm^3 voxels.<br><br>Venous blood samples were collected from participants. Serum NfL concentration was measured using the Human Neurology 4-Plex A assay (N4PA) on an HD-1 Single molecule array (Simoa) instrument according to instructions from the manufacturer (Quanterix, Billerica, MA).<br><br>Personality was assessed based on different subscales of the Personality Assessment Inventory protocol.
Data analysis	Matlab and scikit-learn package from Python were used for analysis.

For manuscripts utilizing custom algorithms or software that are central to the research but not yet described in published literature, software must be made available to editors and reviewers. We strongly encourage code deposition in a community repository (e.g. GitHub). See the Nature Research guidelines for submitting code & software for further information.

Data

Policy information about availability of data

All manuscripts must include a data availability statement. This statement should provide the following information, where applicable:

- Accession codes, unique identifiers, or web links for publicly available datasets
- A list of figures that have associated raw data
- A description of any restrictions on data availability

The neuroimaging data that supports the findings of this study is subject to confidentiality agreement and the patients have not consented to public release of their data. Access to the neuroimaging dataset can be requested to M.C.T. (Carmela.Tartaglia@uhn.ca). GSP features extracted from neuroimaging data and serum NfL levels for HC and ExPro cohorts that support the results in Fig. 2 and Fig. 3 are provided as Supplementary Data 1,2,3, and 4. The age data for HC and ExPro cohorts are available in Supplementary Data 5 and 6. PAI aggression scores for HC and ExPro cohorts are available in Supplementary Data 7 and 8. Cortical thickness data for HC and ExPro cohorts are available in Supplementary Data 9 and 10. Source data files for Fig.2 a,b,c are available in Supplementary Data 12, for Fig. 2 d,e,f are available in Supplementary Data 13, for Fig. 3 a,b,c are available in Supplementary Data 14, and for Fig. 3 d,e,f are available in Supplementary Data 15. Source data for Fig. 4a is available in Supplementary Data 1, 3, and 5. Source data for Fig. 4b is available in Supplementary Data 1,3, and 7. Source data for Fig. 4c is available in Supplementary Data 2 and 10. Source data for Fig. 4d is available in Supplementary Data 2, 4, and 11.

Field-specific reporting

Please select the one below that is the best fit for your research. If you are not sure, read the appropriate sections before making your selection.

☒ Life sciences ☐ Behavioural & social sciences ☐ Ecological, evolutionary & environmental sciences

For a reference copy of the document with all sections, see nature.com/documents/nr-reporting-summary-flat.pdf

Life sciences study design

All studies must disclose on these points even when the disclosure is negative.

Sample size	We analyzed the data collected from 56 subjects.
Data exclusions	One subject each from the HC and ExPro group were excluded from the study since their serum NfL level was more than three deviations from the mean serum NfL level in their respective groups.
Replication	Leave-one-out cross validation was performed for all serum NfL prediction models to establish their prediction performance.
Randomization	The healthy control subjects were recruited from the community. The subjects had no history of neurological disorders (e.g., seizure disorder), systemic illnesses known to affect the brain (e.g., diabetes and lupus), psychotic disorder, or known developmental disorders (e.g., attention deficit disorder, dyslexia) nor any lesions appearing on MRI. The former athletes (ExPro subjects) were former professional football, hockey or boxing athletes with history of multiple concussions.
Blinding	Blinding was not relevant to this study as we focused on extracting cohort-specific features that distinguished between them.

Reporting for specific materials, systems and methods

We require information from authors about some types of materials, experimental systems and methods used in many studies. Here, indicate whether each material, system or method listed is relevant to your study. If you are not sure if a list item applies to your research, read the appropriate section before selecting a response.

Materials & experimental systems

n/a	Involved in the study	
☒	☐	Antibodies
☒	☐	Eukaryotic cell lines
☒	☐	Palaeontology and archaeology
☒	☐	Animals and other organisms
☐	☒	Human research participants
☐	☒	Clinical data
☒	☐	Dual use research of concern

Methods

n/a	Involved in the study	
☒	☐	ChIP-seq
☒	☐	Flow cytometry
☐	☒	MRI-based neuroimaging

Human research participants

Policy information about studies involving human research participants

Population characteristics	The healthy control subjects (number = 20, mean age = 49.38 years, standard deviation = 10.94 years) had no history of

Population characteristics	neurological disorders (e.g., seizure disorder), systemic illnesses known to affect the brain (e.g., diabetes and lupus), psychotic disorder, or known developmental disorders (e.g., attention deficit disorder, dyslexia) nor any lesions appearing on MRI. The former athletes (number = 36, mean age = 50.64 years, standard deviation = 11.36 years) were former professional football, hockey or boxing athletes with history of multiple concussions (mean = 4.14, standard deviation = 1.7). There was no significant difference between the ages or serum NfL levels of the two groups (MannWhitney U tests at 0.05 significance level). One subject each from the HC and ExPro group were excluded from the study since their serum NfL level was more than three deviations from the mean serum NfL level in their respective groups. There was no significant difference between the years of education for the two groups (HC: mean number of years of education = 16.4 years, standard deviation = 1.81 years, ExPro: mean number of years of education = 15.82 years, standard deviation = 1.68 years). Furthermore, no significant difference was observed in the cognitive scores in the contexts of memory, language and visuospatial function for the two cohorts. Differences on inhibitory control, which is an executive function, have been reported previously on this sample (Terpstra et al., 2019).
Recruitment	Healthy control subjects were recruited from the community. The former athletes were former professional football, hockey or boxing athletes with history of multiple concussions.
Ethics oversight	The study was approved by the research ethics boards of the University Health Network.

Note that full information on the approval of the study protocol must also be provided in the manuscript.

Clinical data

Policy information about clinical studies

All manuscripts should comply with the ICMJE guidelines for publication of clinical research and a completed CONSORT checklist must be included with all submissions.

Clinical trial registration	NCT03218332
Study protocol	https://clinicaltrials.gov/ct2/show/NCT03218332?term=tartaglia&draw=2&rank=8
Data collection	Academic Hospital, ongoing study (2012-present)
Outcomes	Biomarkers for possible CTE (Neuropsychological/MRI/genetics/CSF/PET-tau/oculomotor)

Magnetic resonance imaging

Experimental design

Design type	Resting state
Design specifications	All structural and resting state scans were performed on a 3 Tesla MRI Scanner (GE Signa HDx, Milwaukee, WI, USA) with a standard 8-channel head coil. A high resolution T1-weighted images were obtained using inversion recovery fast spoiled gradient echo (IR-FSPGR), with the following parameters: 180 slices with 1 mm thickness; 3 ms echo time (TE); 7.8 ms repetition time (TR); 450 ms inversion time (TI); 15 flip angle; 25.6 cm field of view (FOV); 256×256 matrix size; 1×1×1 mm^3 voxel size. At least one DWI scan was obtained with diffusion gradients applied across 60 spatial directions (b = 1,000 s/mm^2) as well as 10 non-diffusion weighted (B0) scans. The DWI had the following parameters: 2.4 mm thick axial slices, TR = 17,000 ms, FOV = 23 cm, 2.4×2.4 mm^2 in-plane resolution. Prior to the resting-state functional MRI scan, participants were instructed to close their eyes, not think of anything in particular, and to not fall asleep. The scan acquisition was 5 min 8 s using T2^*-weighted echo-planar imaging with the following parameters: TR = 2,000 ms, TE = 30 ms, 64×64 matrix, 20-cm FOV, flip angle = 85, 40 slices, 3.125×3.125×4 mm^3 voxels.
Behavioral performance measures	No task was involved.

Acquisition

Imaging type(s)	Functional, diffusion
Field strength	3 Tesla
Sequence & imaging parameters	See Design Specifications.
Area of acquisition	Whole brain scan.

Diffusion MRI ☒ Used ☐ Not used

Parameters	See Design Specifications.

Preprocessing

Preprocessing software	Diffusion MRI data were processed using the SCRIPTS pipeline with parameters as described therein. Pre-processing involved correction for eddy-currents and head motions artifacts using FSL. After alignment of the co-registered dMRI to the T1 image, fiber tracking was performed using the MRtrix3 package. Fiber orientation estimation was performed using Constrained Spherical Deconvolution, and tracks were seeded from the white-gray matter interface. A propagation mask was applied

through Anatomically Constrained Tractography (ACT) and streamlines were generated using a probabilistic algorithm using second-order integration over fiber orientation distributions (iFOD2) from 10 million seeds (step size 0.5 mm, maximum curvature 45, length 5-250 mm, FOD amplitude threshold 0.1). Streamlines were then selected using Spherical-deconvolution Informed Filtering of Tractograms (SIFT) to improve the fit between streamline reconstruction and the original dMRI image. The connectome weights are defined by the number of tracks going from one area of the parcellation mask to another, using the Desikan-Killiany atlas.

Functional MRI data were processed using fMRIPrep, an open-source pipeline integrating multiple state-of-the-art fMRI tools into a single software suite. Motion artifact correction and denoising were performed using ICA-AROMA, and susceptibility distortion corrections were performed using the SyN "fieldmap-less" correction method implemented in Advanced Normalization Tools (ANTs).

Normalization: dMRI was co-registered to the T1 image.

Normalization template: subject space, T1 image

Noise and artifact removal: Corrections for eddy currents and head motions artifacts in Diffusion MRI were performed using FSL. in Functional MRI images, motion artifact correction and denoising were performed using ICA-AROMA, and susceptibility distortion corrections were performed using the SyN "fieldmap-less" correction method implemented in Advanced Normalization Tools (ANTs).

BOLD time series of length 308 seconds (154 time points) were exported in CIFTI format, and the first 18 seconds were discarded to remove initialization transient artifact. Additionally, the BOLD time series were pre-processed by removal of any linear trends and constant offsets and passed through a band-pass frequency domain filter with range 0.009 Hz - 0.1 Hz.

Volume censoring: *Define your software and/or method and criteria for volume censoring, and state the extent of such censoring.*

Statistical modeling & inference

Model type and settings: Partial least squares regression was used for multivariate regression of GSP features against serum NfL and predictive analysis.

Effect(s) tested: Ranksum statistic, pearson's correlation, partial correlation

Specify type of analysis: ☐ Whole brain ☐ ROI-based ☒ Both

Anatomical location(s): Anatomical locations pertinent to serum NfL were identified for healthy controls and former athletes.

Statistic type for inference (See Eklund et al. 2016): Cluster wise analysis was used on cortical regions based on Desikan-Killiany atlas.

Correction: FDR and permutation tests based correction.

Models & analysis

n/a	Involved in the study
☒	☐ Functional and/or effective connectivity
☐	☒ Graph analysis
☐	☒ Multivariate modeling or predictive analysis

Graph analysis: Weighted graph with weights representing number of axonal connections. Graph signal processing analysis was performed that leveraged eigenmodes of structural connectome to filter BOLD time series signal.

Multivariate modeling and predictive analysis: Partial least squares regression was used with GSP features as predictors and serum NfL as the dependent variable. Linear regression analysis was used with age as predictor and serum NfL as the dependent variable. Moderation analysis was used to characterize the moderation effect of serum NfL on association between right amygdala and GSP feature with age as a covariate.
Mediation analysis was used in the following settings:
a) age as predictor, serum NfL as the dependent variable and GSP feature as mediating variable.
b) pericalcarine thickness as predictor, serum NfL as the dependent variable, GSP features as mediating variable.
c) age as predictor, GSP feature as dependent variable, left choroid plexus volume as mediating variable.
